# Editorial for the Special Issue: “Phage Therapy: A Biological Approach to Treatment of Bacterial Infections”

**DOI:** 10.3390/antibiotics9100721

**Published:** 2020-10-21

**Authors:** Saija Kiljunen

**Affiliations:** Human Microbiome Research Program, Faculty of Medicine, University of Helsinki, 00290 Helsinki, Finland; saija.kiljunen@helsinki.fi

The emergence of antibiotic-resistant bacteria presents a major challenge in terms of increased morbidity, mortality, and healthcare costs. The World Health Organization (WHO) states that antibiotic resistance is one of the biggest threats to global health, food security, and development. One promising alternative to treat infections caused by antibiotic-resistant bacteria is phage therapy, where bacteriophages (phages) are used to combat the pathogens. Phage therapy has a 100 years history, but it has been largely ignored in Western countries after the commercialization of antibiotics. Now, as antibiotic-resistant bacteria are becoming more common, a renewed interest in phage therapy is emerging.

This Special Issue “Phage Therapy: A Biological Approach to Treatment of Bacterial Infections” covers different aspects of phage therapy. The issue altogether includes nine papers, four out of which are research papers, four are reviews, and one is a case study.

Two of the published articles concentrate on the isolation and characterization of phages and on setting up a phage depository. Styles et al., describe the analysis of phages infecting *Acinetobacter baumannii*, which were isolated earlier in hospital wastewater in Thailand. They show that each of the analyzed five phages can infect 22–28% of 150 multiresistant *A. baumannii* strains and that one of the phages clearly reduces the cytotoxic effect of *A. baumannii* on human cells in vitro [1]. Yerushalmy et al., give a detailed report about the establishment of a phage bank of over 300 phages targeting 16 pathogenic bacterial species and describe the protocol they use to isolate and characterize new phages [2].

Interestingly, three articles in the Special Issue concern phage therapy in aquaculture. A review article by Zaczek et al., discusses different aspects of phage application in aquaculture niches from both theoretical and practical points of view. They handle topics such as microbial spoilage of seafood, phage abundance in aquatic systems, and therapeutic application of phages in the fish industry in practice [3]. The two research papers from the group of Lotta-Riina Sundberg approach the topic from slightly different angles. The paper by Almeida et al., shows that after a single addition of a phage into a recirculating aquaculture system, the phage can persist in the system for up to 14 days [4]. Laanto et al., characterize the mechanisms of how *Flavobacterium columnare* becomes phage resistant in a one-month co-culture of the phage and the bacterium in lake water and describe the isolation and analysis of an evolved phage that can overcome the phage resistance in some of the bacterial mutants resistant to the original phage [5].

There are three review articles in the Special Issue that cover human phage therapy from different perspectives. The article by Steven Abedon gives a thorough summary of published information about the combination therapy of phages and antibiotics [6] and Zalewska-Piatek and Piatek describe how phage therapy has been used to treat urinary tract infections caused by *Escherichia coli* [7]. The article by Duplessis and Biswas is divided into two sections, in the first part, the authors provide a literature review about topical phage therapy of chronically infected wounds. In the second part, they describe the preparations they have made to initiate a randomized clinical trial aiming to evaluate the safety and therapeutic efficacy of phage therapy in infected diabetic foot ulcers [8]. 

The last article of the Special Issue is a case report by Rubalskii et al., describing the phage therapy of eight patients having severe bacterial infections related to cardiothoracic surgery. The authors report the eradication of the pathogenic bacteria in seven out of eight patients for whom antibiotic treatments had earlier failed [9].

This Special Issue represents a multifaceted collection of research and review articles that together cover several aspects of phage therapy: setting up a phage collection and the isolation and analysis of phages, the application of phages in the food industry (as represented by aquaculture), and the treatment of different types of human infections. We hope that the articles in this Special Issue will reach a wide audience and provide new information for professionals working on different aspects of phage research. 
